# Measurements of cerebrospinal fluid production: a review of the limitations and advantages of current methodologies

**DOI:** 10.1186/s12987-022-00382-4

**Published:** 2022-12-15

**Authors:** Guojun Liu, Antonio Ladrón-de-Guevara, Yara Izhiman, Maiken Nedergaard, Ting Du

**Affiliations:** 1 Department of Neurosurgery, The Fourth Affiliated Hospital of China Medical University, Shenyang, 110032 China; 2 School of Pharmacy, China Medical University, Shenyang, 110122 China; 3 Center for Translational Neuromedicine, Faculty of Health and Medical Sciences, University of Copenhagen, 2200 Copenhagen, Denmark; 4 Center for Translational Neuromedicine, Department of Neurosurgery, University of Rochester Medical Center, Rochester, NY 14642 USA

**Keywords:** Cerebrospinal fluid, Choroid plexus, CSF production rate, CSF dynamics

## Abstract

Cerebrospinal fluid (CSF) is an essential and critical component of the central nervous system (CNS). According to the concept of the “third circulation” originally proposed by Cushing, CSF is mainly produced by the choroid plexus and subsequently leaves the cerebral ventricles via the foramen of Magendie and Luschka. CSF then fills the subarachnoid space from whence it disperses to all parts of the CNS, including the forebrain and spinal cord. CSF provides buoyancy to the submerged brain, thus protecting it against mechanical injury. CSF is also transported via the glymphatic pathway to reach deep interstitial brain regions along perivascular channels; this CSF clearance pathway promotes transport of energy metabolites and signaling molecules, and the clearance of metabolic waste. In particular, CSF is now intensively studied as a carrier for the removal of proteins implicated in neurodegeneration, such as amyloid-β and tau. Despite this key function of CSF, there is little information about its production rate, the factors controlling CSF production, and the impact of diseases on CSF flux. Therefore, we consider it to be a matter of paramount importance to quantify better the rate of CSF production, thereby obtaining a better understanding of CSF dynamics. To this end, we now review the existing methods developed to measure CSF production, including invasive, noninvasive, direct, and indirect methods, and MRI-based techniques. Depending on the methodology, estimates of CSF production rates in a given species can extend over a ten-fold range. Throughout this review, we interrogate the technical details of CSF measurement methods and discuss the consequences of minor experimental modifications on estimates of production rate. Our aim is to highlight the gaps in our knowledge and inspire the development of more accurate, reproducible, and less invasive techniques for quantitation of CSF production.

## Introduction

The past decade has seen burgeoning interest in the classical topic of cerebrospinal fluid (CSF) production and transport. The recent findings that CSF transport mediates the export from the brain of proteins implicated in neurodegenerative diseases has inspired a range of clinical and preclinical studies [1–7]. Furthermore, CSF transport has attracted new interest in the circadian rhythm and sleep research communities, given that rate of solute outflow follows a diurnal cycle, with a maximal rate occurring during sleep [8]. In this regard, sleep disturbances and deteriorated sleep quality have been identified as predisposing factors for age-dependent cognitive decline and neurodegenerative diseases including Alzheimer’s disease, Parkinson’s disease, and multiple sclerosis [9]. Indeed, the clearance rate for intracerebral amyloid-β more than doubles during sleep in mice [8] In humans, one night of sleep deprivation increases the amyloid-β concentration in the CSF [10], and the binding density of amyloid-β in subcortical brain regions has an inverse correlation with the reported number of night sleep hours [11]. Short sleep duration during midlife is associated with a higher risk of dementia later in life, independently of sociodemographic, behavioral, cardiometabolic, and mental health factors [12]. Moreover, subjects with permanent night work have a higher risk of dementia than controls [13]. For obvious reasons, CSF production is a prerequisite for CSF efflux and associated metabolite clearance. It is therefore unfortunate that our knowledge of basic aspects of the CSF production process and rate remain limited. For example, a century after Henry Cushing’s proposal of the third circulation, the anatomic sources of CSF production as well as total CSF production rates are both debated [14]. Furthermore, the physiological regulators of ion and water transporters subserving CSF production have yet to be established [15, 16]. Over the past 60 years, multiple research groups have developed various methodologies to quantify the absolute rate of CSF production, with varying results. In this review, we summarize the existing methodologies, aiming to outline their main differences, advantages, and disadvantages. We hope to encourage further research and refinement of the existing techniques, enabling the more accurate measurement of CSF production in preclinical and clinical setups using minimally invasive procedures.

## The history of cerebrospinal fuild

**Fig. 1:**
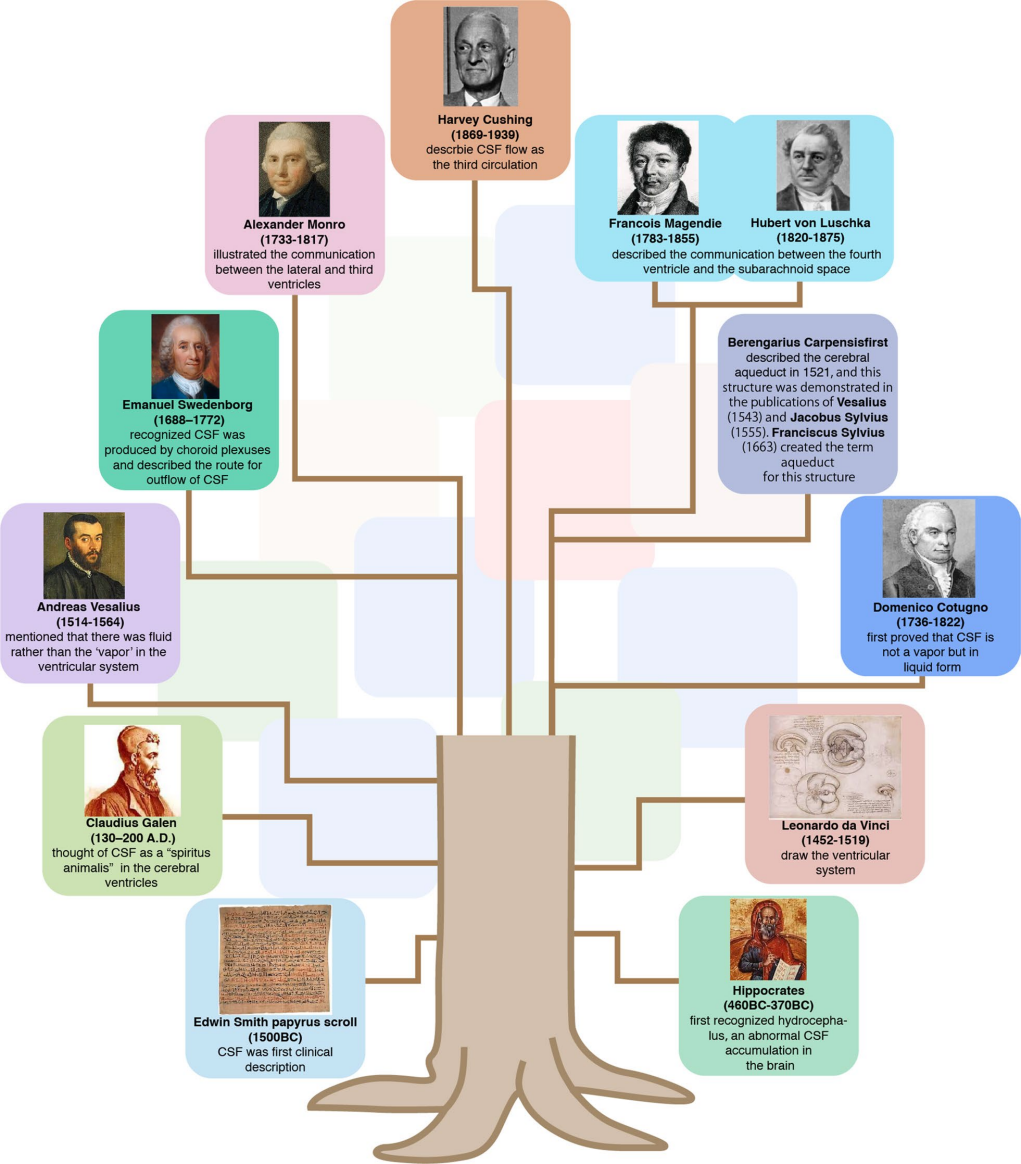
Timeline tree of important discoveries pertaining to CSF. Timeline tree of significant events and discoveries pertaining to the choroid plexus and CSF production. The branches illustrate the passage of time, which spans over millennia, beginning with the first clinical description of CSF in an ancient Egyptian papyrus dating from 1500 BC, the first confirmation of CSF as a liquid during the Renaissance, and the modern description of CSF transport as the third circulation by Harvey Cushing

The first clinical description of CSF is present in an Egyptian papyrus scroll discovered in 1862 by Edwin Smith [17]. The papyrus dates to the Middle Kingdom ending 1600 BC, but its description of the CSF and intracranial pulsations may have originated in the earliest dynastic times dating back to 3000 BC. The great physician Hippocrates (*Hippokrátēs ho Kôios*; c. 460–370 BC) first recognized hydrocephalus in animals and humans [18], which is now understood to represent an abnormal accumulation of CSF within the cranium. Later on, Claudius Galen of Pergamon (130–200 A.D.) thought of CSF as a “*spiritus animalis*” located in the cerebral ventricles that provides the entire body with energy and vitality [15, 19, 20]. During those early times, anatomists believed that the subarachnoid space was filled with this vapor, which they considered to condense to water after death due to the rapid decrease in body temperature [21]. According to such a model, measurement of the CSF volume in living brain would not be possible. In the Italian Renaissance, Leonardo da Vinci (1452–1519) made huge contributions to cerebral anatomy, notably through his careful drawings of the ventricular system, as visualized by the injection of liquid wax into the ventricles, which are apt to be drained of CSF during organ collection [22]. Andreas Vesalius (1514–1564) demonstrated the errors in Galen’s conception of CSF, and described that the brain and its fluid spaces are confined within the rigid cranial vault. Abandoning Galen’s vapor, Vesalius contended that fluid was the main constituent of the ventricular system [23]. Continuing the Italian Renaissance tradition of medical research, Domenico Cotugno (1736–1822) made the first proof that CSF is indeed not a vapor that condenses *post mortem*, based on his observations in fresh cadavers. While attempting to collect the CSF, he held the cadaver in the upright position and avoided tearing the dura mater during the dissection of the cranium, thus obtaining better recovery of the fluid [21]. By this means, Cotugno estimated the volume of human CSF to be three Neapolitan ounces, or roughly 80 mL [15, 24]. This CSF volume estimate is only one half of the current generally accepted estimate of ~ 150 mL in adult male humans [25], a discrepancy that is best explained by uptake of CSF by neural cells or the brain parenchyma after cardiac arrest [26]. About the same time as Cotugno was working on cadavers, the Swedish natural scientist and mystic Emanuel Swedenborg (1688–1772) recognized that CSF was produced by the choroid plexus (CP). He first described the outflow of CSF arising from the roof of the fourth ventricle, and made it clear that CSF surrounds the medulla oblongata and flows caudally through the foramen magnum of the skull to run down the posterior surface of the spinal cord. Swedenborg was also the first to observe and describe the central canal of the spinal cord [27]. Indeed, the Italian surgeon Berengarius Carpensis had described the cerebral aqueduct as the anterior point of CSF entry to the spinal cord as early as 1521 [28, 29], and this structure was presented in the publications of Vesalius (1543) [28] and the Frenchman Jacobus Sylvius (1555) [30], whose Dutch successor Franciscus Sylvius designated the term aqueduct for this structure in 1663. The Scottish physician Alexander Monro (1733–1817) gave a first description of an enlarged cerebral aqueduct in the context of hydrocephalus in 1764 [31], and illustrated the communication of fluid between the lateral and third ventricles in 1783 [32]. The French physiologist Francois Magendie (1783–1855) and the German anatomist Hubert von Luschka (1820–1875) went on to describe the communication of fluid between the fourth ventricle and the subarachnoid space at the beginning of the nineteenth century [33]. In 1925, the American neurosurgeon Harvey Cushing published his milestone paper “The third circulation and its channels”, wherein he established the basis of the modern understanding of CSF physiology [34]. Cushing suggested that CSF flows through the ventricles, cisterns and subarachnoid space, and is reabsorbed into the blood at the arachnoid villi and granulations. This principle of CSF circulation has ever since been the major pillar for understanding CNS fluid flow, albeit that recent studies have suggested a more complex pattern of CSF transport. The timeline of important discoveries pertaining to CSF are summarized in Fig. 1.

## Methodologies used for measurements of CSF production

### Comparison of relative cell volumes between the vein of choroid plexus and aorta

**Fig. 2:**
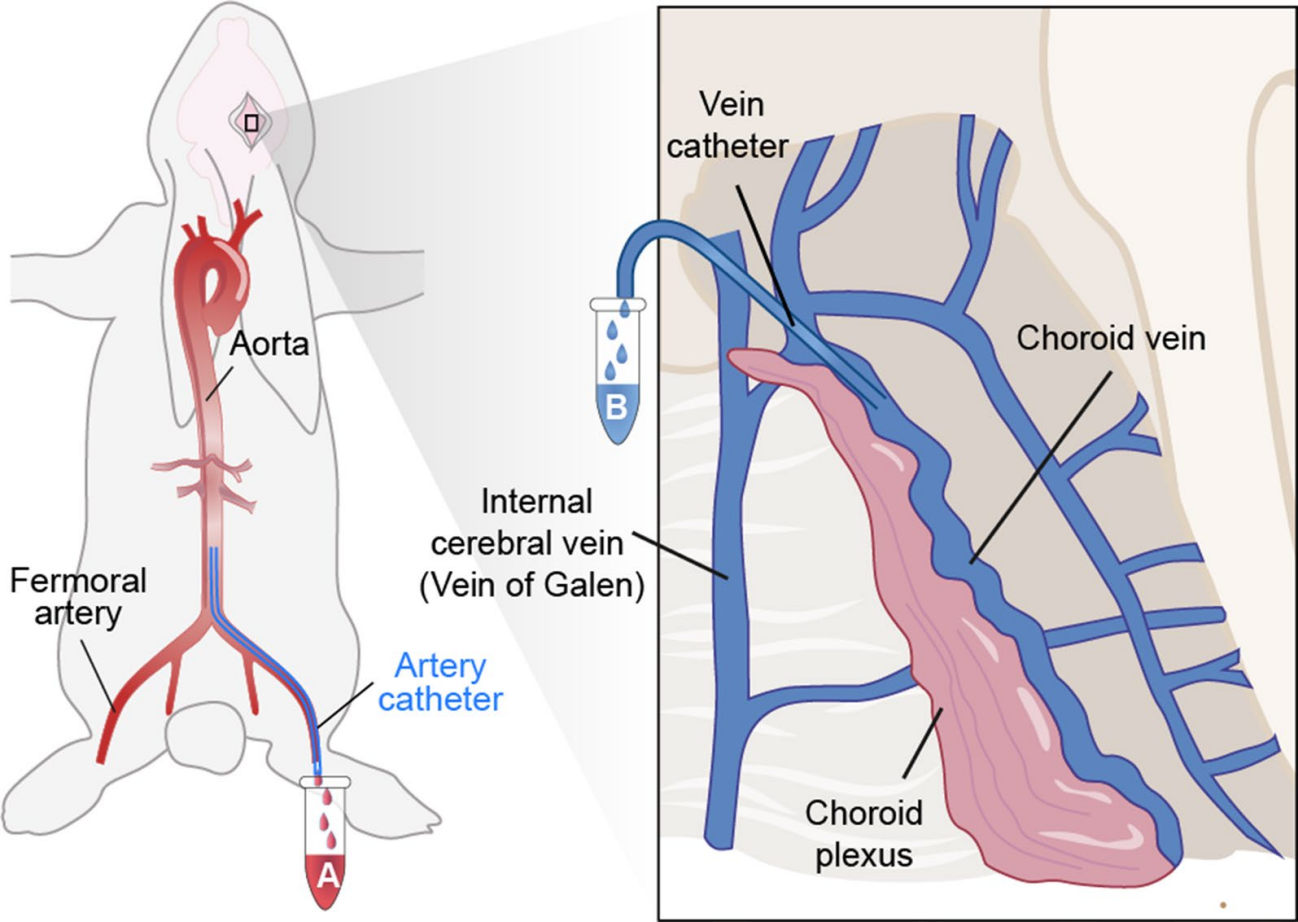
Comparison of Relative Cell Volumes Between the Vein of Choroid Plexus and Aorta. A catheter is inserted into a rat femoral artery and pushed into the aorta to collect aortic blood in Tube A. A micropipette is introduced into the anterior part of the main choroidal vein to collect venous blood in Tube B. The hematocrit is measured in aortic blood (Tube A) and in venous blood of the choroid plexus (Tube B). The CSF production rate is calculated as being equal to the relative volume loss of the plasma compartment during the transit of blood through the choroid plexus

In 1963, Keasley Welch published a method for measuring the CSF production rate [35], taking advantage of the large choroid plexus vein in the lateral ventricle of the rabbit brain, from which it is possible to collect venous blood, as shown in Fig. 2. The Welch method is based on the theoretical principle that relative volume loss of the plasma compartment during the transit of blood through the CP is equivalent to CSF production rate. Thus, the method calls for measuring the relative blood cell volume, or hematocrit, in aortic arterial blood and in venous blood of the CP [36]. The so-calculated CSF production rate by the rabbit choroid plexus was 0.37 μl/min/mg [35]. Given of the mean weight of the rabbit choroid plexus (23.4 mg), this predicts a total production rate of 8.65 μL/min. This CSF production rate is some 40% less than the corresponding result of 12.1 μL/min according to the (see “Indirect Perfusion Method (Dilution Method)” Section) [37]. The discrepancy of the two observations may reflect underestimation by Welch’s method due to the exclusion of extrachoroidal resources, or overestimation by the indirect method based on tracer infusion, as discussed below. Focusing on Welch’s method, we note several limitations: (1) A craniectomy is required to open the dura mater, which causes a decrease of the intracranial pressure (ICP) that affects cerebral blood flow. Exposing the CP in the lateral ventricle called for removal of a portion of cerebral cortex, rendering it a highly invasive procedure. (2) The prerequisite for CSF flow calculation lies in assuming that the hematocrit of blood in the choroid plexus arteries matches that in aorta. However, as addressed by Cserr, this assumption is likely to be violated [38]. A loss of plasma volume due to hematocrit changes during the transit of blood across the capillary bad may occur in the brain parenchyma as well as in the CP, which likely imparts some unreliability to Welch’s method.

### Extracorporeal perfusion of choroid plexus

**Fig. 3:**
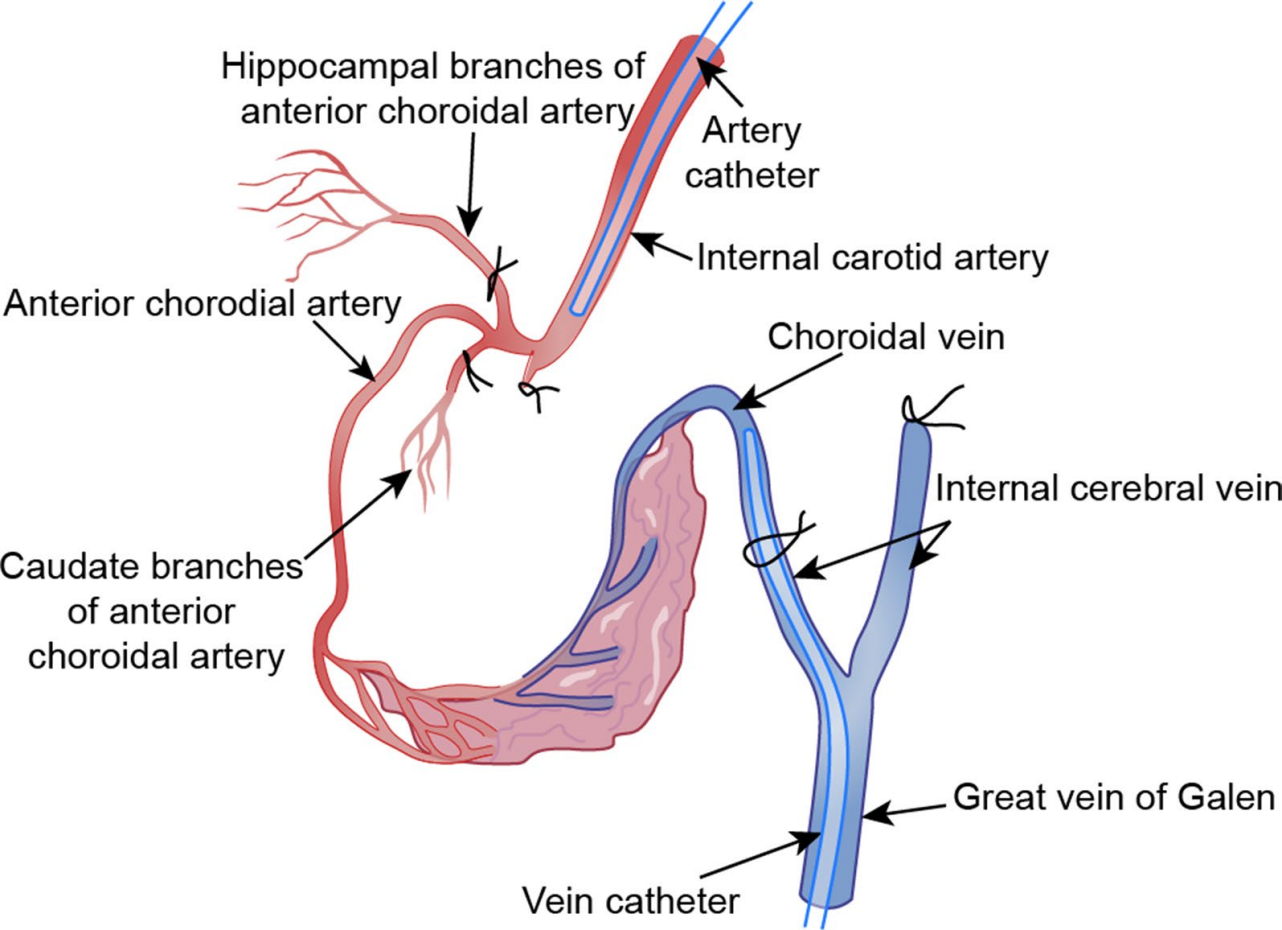
Catheter Placement Method in the Vascular System of the Choroid Plexus in Sheep Brain. The midline great vein of Galen is cannulated with flexible silastic tubing and the catheter is advanced into internal cerebral vein on the side of the perfused plexus, while another internal carotid artery branch and the anterior choroidal artery branches are occluded by bipolar electrocoagulation. Donor sheep blood containing 10% dextran is perfused into a silastic tube, which cannulates the internal carotid artery, and outflow is collected from the catheter in the internal cerebral vein. The difference of hematocrit between the anterior chorodial artery and internal cerebral vein is taken to represent CSF production rate in vitro. Modified from Pollay et al. [39]

Pollay modified the Welch method in an effort to circumvent the bias due violation of assumption of lacking hematocrit differences between aortic and choroid plexus arteries [39]. Pollay used sheep as experimental animals because the main part of their CP is supplied by a single branch of the anterior choroidal artery [14]. Also, venous blood sampling is facilitated by the veins of the CP in the foramen of Monro, which are the largest choroidal veins in sheep brain. The special configuration of the ovine choroid plexus vascular supply in amenable to sampling of the choroidal vein following perfusion with a Ringer-lactate solution via the anterior choroidal artery [14]. In Pollay’s method, the sheep were decapitated, and the skull dissected. The blood was then cleared from the cerebral vessels by perfusing cold Ringer-lactate solution into the internal carotid arteries through a Silastic catheter. The hippocampal and caudate branches of the anterior choroidal arteries [14] were occluded, and the great vein of Galen was cannulated as observed in Fig. 3. The catheter was then advanced into the internal cerebral vein. All the veins draining into the internal cerebral vein and great vein of Galen were then ligated using a suture. A perfusate prepared by diluting whole blood in a normal saline solution containing low molecular weight dextrans (10% solution) entered the anterior choroidal artery and was collected from the internal cerebral vein. The CSF production rate was calculated based on the changes of hematocrit values between the anterior choroidal artery and internal cerebral vein sides. This approach gave a CSF production rate of 0.13 μL/mg/min in sheep brain. For the sake of comparison, the CSF production rate of goat brain according to indirect perfusion method of Pappenheim (described in “Indirect Perfusion Method (Dilution Method)” Section below) was 0.3 μL/mg/min, corresponding to 150 μL/min, given the estimated 500 mg total choroid plexus weight [40]. The relatively low estimate obtained with extracorporeal perfusion of CP in sheep may reflect certain technical aspects of the Pollay approach: (1) The CP is vulnerable to hypoxic damage during the ten minute interval between decapitation and arrival of artificial arterial-venous perfusion; (2) The volume of venous blood collected is small, thereby reducing the accuracy of the calculations; (3) During the perfusion, ICP is low and unstable [38]. Due to these disadvantages, the Pollay method is no longer used.

### Roentgenographic method (air replacement method)

**Fig. 4:**
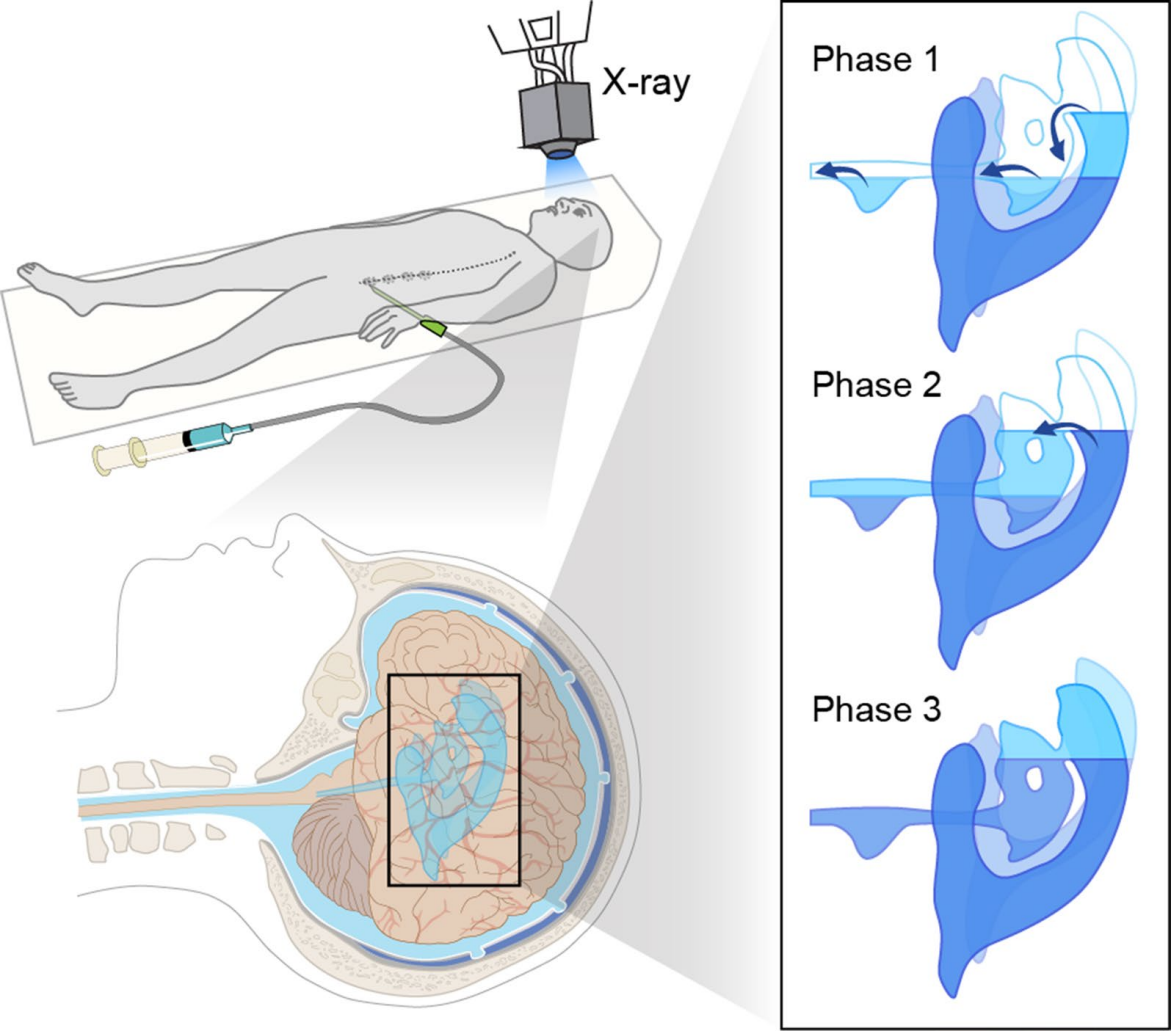
The Three Air Displacement Phases within the Ventricular System. The patient is in the supine position when circa 80 mL CSF is drained via a lumbar puncture followed by its replacement with air. Sequential roentgenographic imaging of the ventricles, the aqueduct of the third ventricle, and the fourth ventricle are acquired. Phase 1: the fluid level rises as the newly formed CSF accumulates in the lateral ventricles. The air is displaced into the third ventricles. Phase 2: the CSF flows into the aqueduct and the fourth ventricle when the lateral ventricles are filled with CSF to the level of the foramen of Monro. Phase 3: the CSF level elevates when the fluid level reaches the posterior margin of the foramen of Monro as the air is re-absorbed. Modified from Deck et al. [42]

The roentgenographic method of CSF production was developed by Potts and collaborators in the 1960s [41–43]. They recruited patients suffering from lesions or suspected lesions in the brain who were referred for pneumoencephalography, a now obsolete radiological practice. The procedure involved draining CSF via a lumbar puncture followed by injection of a corresponding volume of air. The patient’s head was then shaken gently to allow as much air as possible to rise into the upper lateral ventricle [41]. During the measurement of CSF production, the head was placed in the brow-up position, to ensure that the air would be released from the lateral ventricles through the three phases as displayed in Fig. 4. This would assist in quantifying the newly formed CSF during the period of observation. Serial lateral (from the side of the object) and anteroposterior (from front to back) roentgenograms (X-ray) and tomograms (a selected layer of the body) were taken at specific time intervals and with fixed, predetermined magnifications to visualize the displacement of air by newly-produced CSF; the changing cross-sectional area in serial roentgenograms of the lateral ventricle at the site of the ascending fluid level indicated the increasing CSF volume. The average volume production by pneumoencephalography was only one-quarter of the volume estimated using the lumbar subarachnoid drainage method, perhaps due to the invasive nature of pneumoencephalography [42]. Potts then modified the method for dog experiments in which air was substituted by Lipiodol (ethiodized poppy seed oil), or Pantopaque (Iofendylate; ethyl iodophenylundecylate) radiological contrast agents [44]. The dogs were placed in the supine position, and both lateral ventricles were punctured by needles. Then, the CSF in the lateral ventricles was replaced by air above and by Pantopaque agent below, such that the air-Pantopaque boundary lay close to the intraventricular needles. As a small quantity of CSF progressively accumulated at the boundary of the air and Pantopaque layers, it was gently collected from each lateral ventricle at regular time intervals, while taking pains to avoid removal of either air or Pantopaque medium. Using these roentgenographic methods, the production of CSF in both lateral ventricles was estimated to be 70 μl/min for humans [42], and 4–7 μl/min for dogs [43]. The CSF production rate volumes for this method fall considerably below corresponding results by the MRI method in humans or the indirect method in dogs, as described below (Table 1). There are several possible reasons for these discrepancies: (1) Air may be absorbed in the ventricles and subarachnoid space, such that the decline in air volume may not be equal to the volume of CSF production; (2) Due to pneumatic inflation, the ventricle size may increase when air replaces CSF [45]; (3) The contrast materials used are toxic and could cause edema, with a negative impact on CSF production by the CP [46]. (4) ICP was perturbed during the CSF drainage.

**Table 1:** Measurement of cerebrospinal fluid production rate in different species

Species	Type of methods	Site of CSF collection	Rate of CSF production	References
Human				
	Roentgenographic method	Both lateral ventricles	71 μL/min	Deck and Potts [42]
	Indirect perfusion method (children)	All ventricles (lumbar subarachnoid)	370 μL/min	Rubin et al. [75]
	Indirect perfusion method (children)	All ventricles (lumbar subarachnoid)	350 μL/min	Cutler et al. [77]
	Direct method (CSF withdrawn)	All ventricles (lumbar subarachnoid)	319 μL/min	Masserman [84]
	MRI	Both lateral ventricles, 3rd ventricle	300 μL/min	Huang et al. [104]
	MRI	Both lateral ventricles, 3rd ventricle	600 μL/min	Piechnik et al. [105]
	MRI	Both lateral ventricles, 3rd ventricle	1200 μL/min	Penn et al. [106]
	MRI	Both lateral ventricles, 3rd ventricle	680 μL/min	Gideon et al. [107]
	MRI	Both lateral ventricles, 3rd ventricle	740 μL/min	Yoshida et al. [108]
	ASL-MRI (Arterial spin labeling magnetic resonance imaging)	Both lateral ventricles	2.7 μL/min	Evans et al. [130]
Calf				
	Indirect perfusion method	All ventricles	290 μL/min	Calhoun et al. [199]
Sheep				
	Extracorporeal perfusion of Choroid Plexus	Choroid Plexus of later ventricle	0.13 μL/min/mg (Relation to wt. choroid plexus)	Pollay et al. [39]
Goat				
	Indirect perfusion method	All ventricles	150 μL/min	Pappenheimer et al.[40]
	Indirect perfusion method	All ventricles	160 μL/min	Heisey et al. [59]
	Indirect perfusion method	All ventricles	181.35 μL/min	Pollay and Curl [37]
Dog				
	Roentgenographic method	Both lateral ventricles	4 ~ 7 μL/min	Potts and Bergland [43]
	Indirect perfusion method	All ventricles	65 μL/min	Sato et al. [147]
	Indirect perfusion method	All ventricles	53 μL/min	Oppelt ea al. [200]
	Indirect perfusion method	All ventricles	46.7 μL/min	Vela AR et al. [60]
	Direct method	All ventricles	30 μL/min	Riser [79]
	Direct method	All ventricles	50 μL/min	Bering [148]
Cat				
	Indirect perfusion method	All ventricles	15 μL/min	Oreskovic [149]
	Direct perfusion method	Both lateral ventricles and 3rd ventricle	12.1 μL/min	Flexner and Winters [80]
Rabbit				
	Comparison of relative cell volumes between the vein of choroid plexus and aorta	Choroid Plexus of both later ventricles and 4th ventricle	0.37 μL/min/mg (Relation to wt. choroid plexus)	Welch [35]
	Indirect perfusion method	All ventricles	12.67 μL/min	Pollay and Curl [37]
	Indirect perfusion method	All ventricles	12.1 μL/min	Pollay [201]
Rat				
	Indirect perfusion method	All ventricles	3.38 μL/min	Chodobski et al. [150]
	Direct perfusion method	Both lateral ventricles and 3rd ventricle	0.39–1.40 μL/min	Kaimy et al. [82]
Mouse				
	Indirect perfusion method	All ventricles	0.325 μL/min	Rudick et al. [151]
	Direct perfusion method	Both lateral ventricles and 3rd ventricle	0.090 μL/min	Liu et al. [66]
Shark				
Dogfish	Indirect perfusion method	All ventricles	4 μL/min	Oppelt et al. [146]
Nurse sharks	Indirect perfusion method	All ventricles	5 μL/min	Oppelt et al. [146]
Lemon sharks	Indirect perfusion method	All ventricles	4 μL/min	Oppelt et al. [146]

### CSF extraction technique from the surface of choroid plexus

**Fig. 5:**
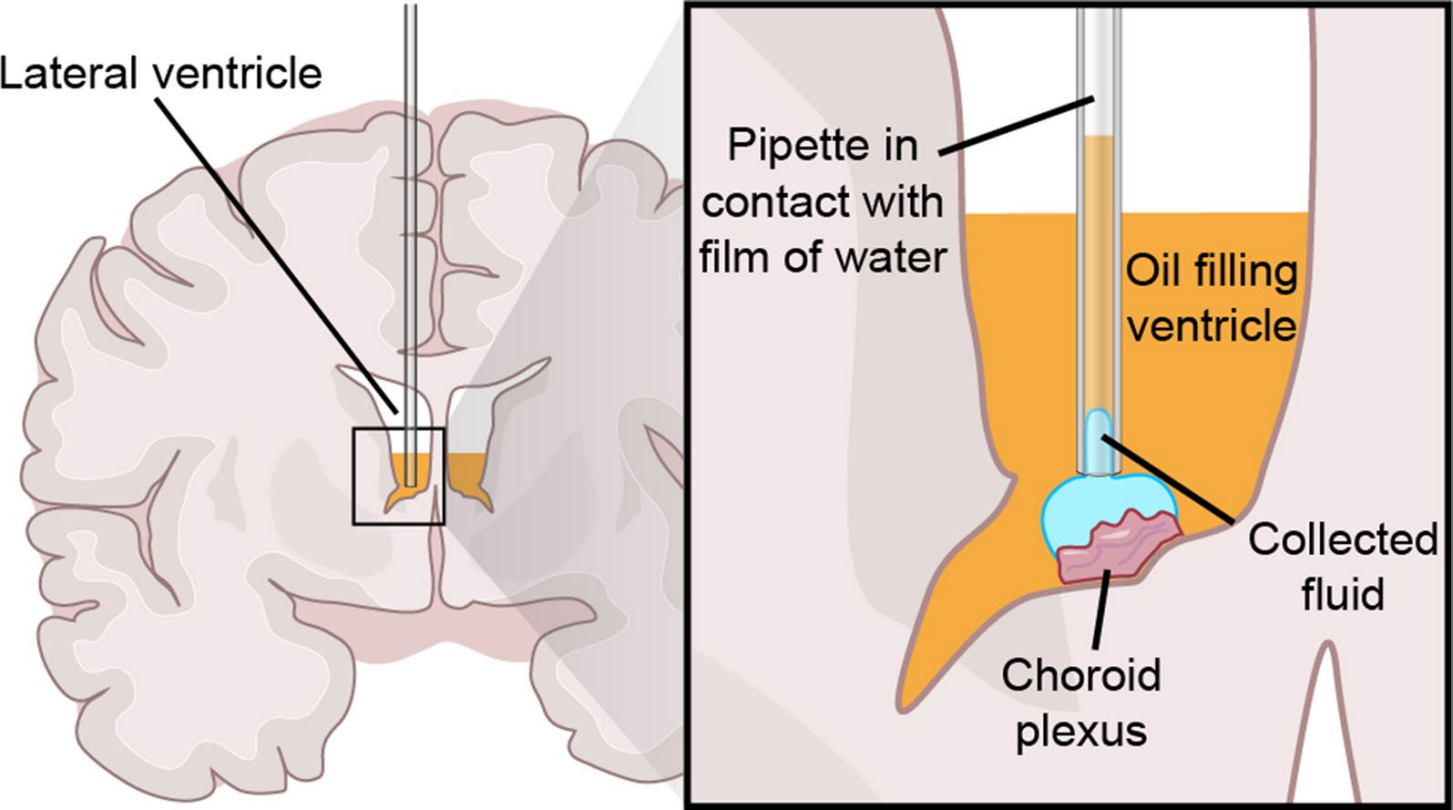
CSF Extraction Technique from the Choroid Plexus. The lateral ventricle is filled with oil and a pipette is placed in close contact with the choroid plexus while the oil–water meniscus moved upwards into it. Based on the property that water has greater affinity for the charged glass surface, the CSF can enter the tube and the level of oil would then rise. The CSF production rate is calculated according to the change in height of the liquid per unit time. Modified from de Rougemont et al. [47]

In this method developed by de Rougemont et al. [47], newly formed CSF is harvested to quantify the CSF production rate. The layout of the technique is shown in Fig. 5. The scalp and the skull of an anesthetized cat was opened to expose the corpus callosum. A blunt dissection was made until the roof of the lateral ventricle was penetrated. The CSF was partially drained from the cisterna magna to empty the ventricles, and then the CSF newly released into the lateral ventricle was immediately displaced with warm ethyl iodophenylundecylate (Pantopaque oil) introduced via a syringe until the fluid surface was about 5 mm above the CP. Next, a fine glass pipette was inserted in the lateral ventricle with the tip just reaching the aqueous film overlying the CP. The upper end of the pipette was open so that the oil could move freely up into the pipette. Because of the greater affinity of the aqueous solution for the glass surface, fluids exuding from the cut corpus callosum or fluid remaining on the walls of the ventricles tended to rise to the surface rather than descending towards the CP. As such, the newly secreted CSF could enter the pipette without the application of suction, and came to occupy an increasingly long segment of the lower part of the pipette [47]. The accumulation of CSF secreted from the CP was measured as a function of time, allowing for the estimation of CSF production rate (μL/min). Furthermore, the electrolyte composition of CSF secreted from the CP was quantified [48, 49]. We note several possible limitations of this method: (1) Hydrostatic pressure building up within the pipette might artifactually increase fluid collection and possibly change its electrolyte composition; (2) The application of Pantopaque oil may change the local CO_2_ tension [47], which in turn may alter production of CSF [50, 51]; (3) This approach is highly invasive and Pantopaque oil is a toxic compound eliciting an inflammatory response in the CP [46]; (4) Also, ICP will drop when the skull is opened.

The many side-effects of the CSF extraction technique have rendered it obsolete, as likewise with the classical approaches described above in “Comparison of Relative Cell Volumes Between the Vein of Choroid Plexus and Aorta” Section to “CSF Extraction Technique from the surface of Choroid Plexus” Section. However, an important lesson to be garnered from these studies is that it is technically possible to harvest newly formed CSF from the ventricles, which affirms the proposition that CP and extrachoroidal sources draining into the ventricles are the main sources of CSF.

### Indirect perfusion method (dilution method)

#### The indirect method for measuring CSF production rate

**Fig. 6:**
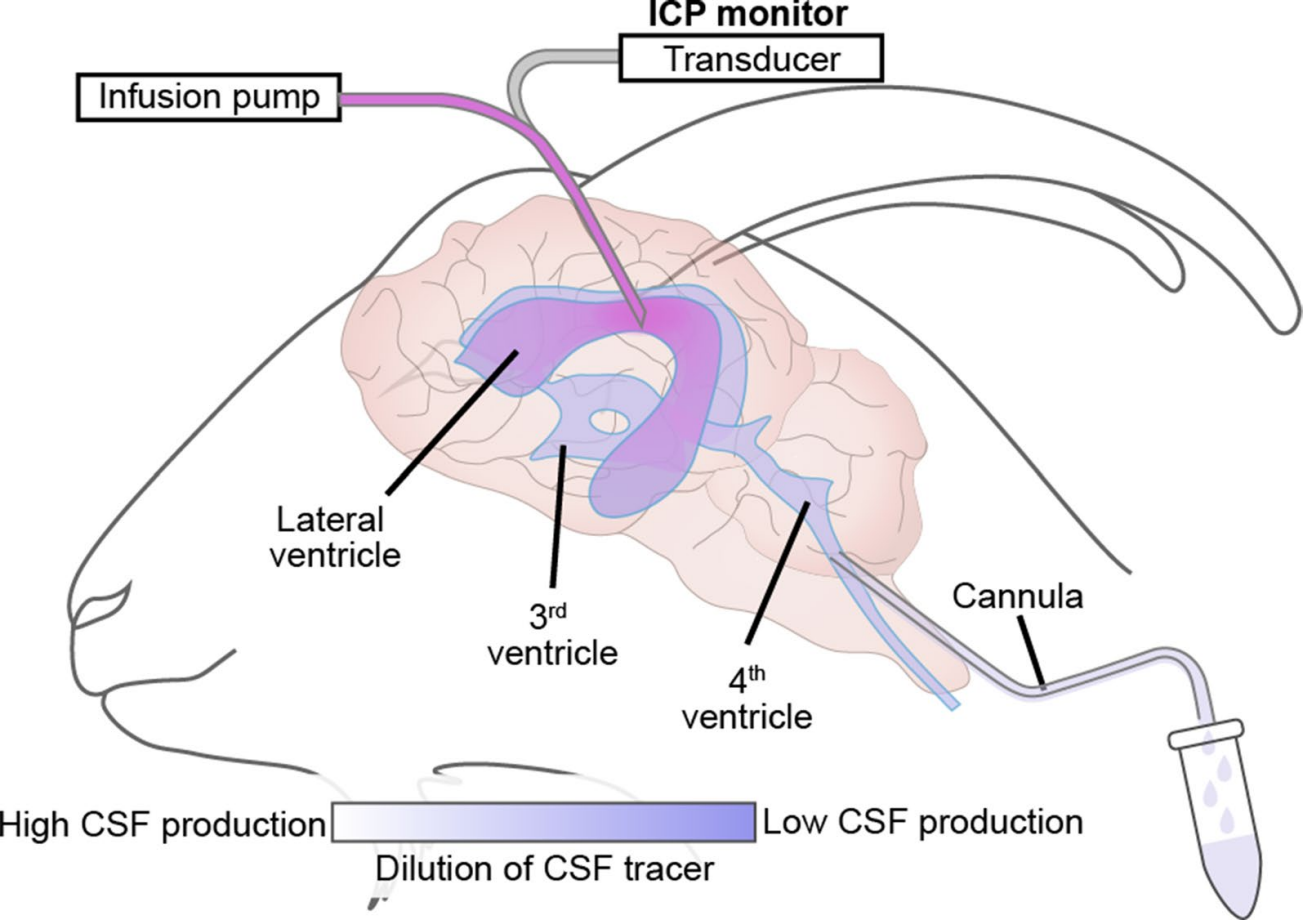
The Procedure used for the Ventriculo-cisternal Perfusion in Goat Brain. A cannula is placed in the left lateral ventricle, and aCSF containing a tracer is perfused into the lateral ventricle. ICP is monitored simultaneously. Another cannula is placed in cisterna magna to collect CSF mixed with the aCSF injected into the lateral ventricle. The relative changes in tracer concentration between what is injected into the lateral ventricle and what is collected in the cisterna magna are used to calculate the CSF production rate. Modified from Oreskovic and Klarica [61]

Historically, the indirect perfusion method is by far the most commonly used technique for quantifying the CSF production rate [52–58]. In 1962 Pappenheimer [40] and Heisey [59] presented this pioneering method in which anesthetized goats were placed in the prone position and operated to gain access to the ventricular system. The indirect perfusion method entails implanting one cannula into the lateral ventricle and another in the cisterna magna, as shown in Fig. 6. Artificial CSF (aCSF) containing a tracer (often insulin or dextran) is perfused into the lateral ventricle cannula and collected from the downstream cisterna magna cannula. The CSF production rate is calculated from the tracer concentration in the CSF collected from the cisterna magna relative to its concentration in the perfused aCSF. According to Heisey’s theory, the CSF production rate was calculated by using the equation [60]:

$$V_{f} = \frac{{V_{i} \times \left( {C_{i} - C_{o} } \right)}}{{C_{o} }},$$

$V_{i}$: rate of CSF production (mL/min), $V_{i}$: rate of infusion (mL/min), $C_{i}$: infused tracer concentration, $C_{o}$: outflowed tracer concentration.

Following the same tracer dilution principle as in the indirect perfusion method, there have been a number of technical modifications, which include ventriculo-ventricular, ventriculo-aqueductal, and ventriculo-lumbar perfusions [36].

The accuracy of the indirect perfusion method for calculating the CSF production rate is based on the validity of several assumptions: (1) The CSF is produced only within the ventricles, such that only ventricular CSF dilutes the tracer; (2) The walls of the ventricles form a barrier against dispersion of tracer into the brain tissue; (3) The infusion rate of the tracer and the elevation of the brain do not influence the production of CSF; (4) The temperature and osmolarity of the aCSF are without effects on the CP secretion of CSF. Several prior reports have noted that this procedure is inaccurate and provides variable results [61]. Specifically, many publications have noted that the CSF tracer indeed enters the brain parenchyma [62–65]. Not just tracers of small molecular size, such as Evans blue (0.96 kDa), but also tracers of large molecular size, like TRITC-dextran (155 kDa), do leak into the neuropil during intraventricular infusion [66]. Unaccounted loss of tracer will be misinterpreted to give an overestimation of CSF production. Furthermore, although most studies agree that the CP represents the primary source of CSF [16, 35, 51, 67, 68], several lines of work show that  ~ 10–20% of the total CSF volume is produced by extra-choroidal pathways that do not drain into the ventricles [69]. The indirect method includes extrachoroidal CSF production that drain into all four ventricles for calculating the rate of CSF production, such that it does not discriminate between contributions of the CP versus extrachoroidal sources. Furthermore, the rate of tracer infusion in the indirect approach perturbs the CSF production [70], with a higher tracer infusion rate resulting in lower calculated CSF production rate. Also, the head elevation in the experimental setup decreases ICP and cerebral perfusion pressure [71], which will influence the production of CSF [72]. The infusion of a large volume of aCSF into the lateral ventricle will tend to cool the ventricular volume and consequently modify CSF production [66]. The osmolality of the infused fluid is also a major determinant of the CSF production rate because low osmolarity reduces the amount of CSF collected, while hyperosmotic solutions increase the volume [73, 74].

#### The indirect method used in human subjects

After Pappenheimer [40] and Heisey [59] had designed and developed the indirect method in the goat model, Rubin et al. were the first to apply the method in humans [75, 76]. In particular, they applied the technique of CSF perfusion intraoperatively during surgical resection of cerebral neoplasms, thus obtaining rates of CSF production rate in adult patients with malignancies of the central nervous system. The aCSF perfusion solution containing the radioactive tracer carboxy-^14^C inulin was perfused in the lateral ventricle and outflow was collected from the lumbar subarachnoid space; inflow rate and volume were determined by a photoelectric drop counter, whereas the outflow solution was collected from a lumbar puncture and determined volumetrically or gravimetrically [75]. The CSF production rate was then calculated using the equation in part “The indirect method for measuring CSF production rate” Section, with typical results close to 0.37 mL/min in human patients. Another research group extended the method to pediatric patients, in whom the data were obtained during perfusion of chemotherapeutic agents by the ventriculo-lumbar route; the production rate in children was 0.35 mL/min, thus matching that in adults [77]. Use of the indirect method in human research has the same limitations as listed above for animal experiments.

### Direct measurements of CSF production

Direct and indirect measurement methods differ principally in that CSF is collected in the lateral ventricles for the direct method, but in cisterna magna for the indirect method. The surgical procedure for the two methodologies is similar with respect to approach and invasiveness. A defining difference is that only the indirect method for measuring CSF production method requires tracer injection and the indirect method is therefore associated with additional complications that can artificially alter the calculated CSF production rate.

#### Animal studies

**Fig. 7:**
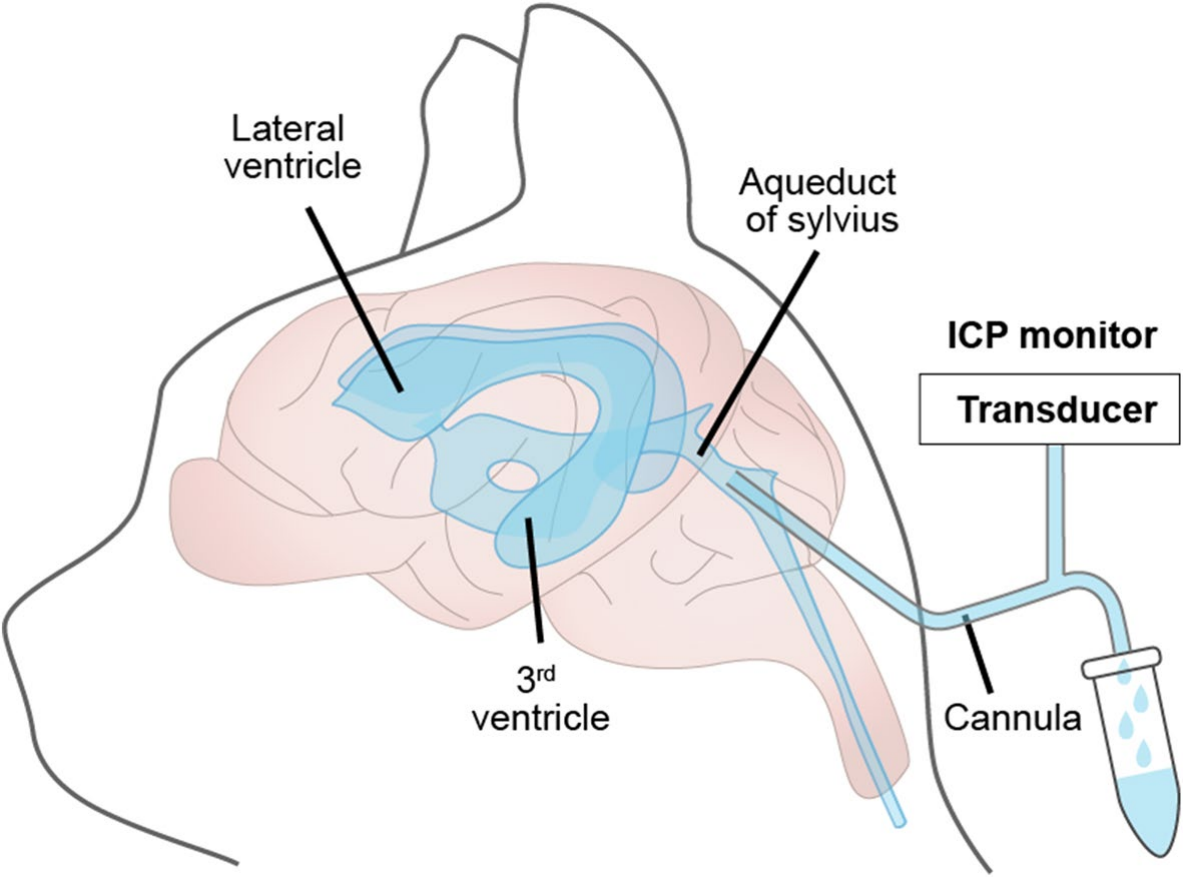
Direct CSF Production Measurement in Cats. A cannula is placed into the aqueduct of Sylvius through the tunnel inside the cerebellar vermis, the outer surface of the cannula is covered by cyanoacrilic gel glue to prevent CSF leakage and the entry of air. The tip of extracranial part of the cannula is used to collect the CSF and measure the ICP

The readiest approach to collect CSF is by direct sampling from the subarachnoid space either in the cisterna magna or via a lumbar puncture. However, direct collection of CSF interferes with ICP, and only some of the CSF will be collected, because it will continue to drain from the CNS by its normal efflux routes during the sampling procedure [78]. In an early application of the direct method, Riser (1929) inserted a cannular into the cisterna magna of a dog weighing 10 kg, in which the apparent CSF production rate was approximately 30 μL/min [79], thus comparable upon body weight scaling to typical findings in human. Flexner and Winters described a direct method of CSF extraction by implanting a cannula into the cerebral aqueduct of the cat, physically blocking the CSF outflow through the aqueduct, and draining the CSF from the cannula [80], as shown in Fig. 7. However, this method suffers from the shortcoming that a negative pressure is required in the collection tube to maintain a regular CSF drip. The application of negative pressure might provoke drainage of fluid that would not normally enter the aqueduct [81]. Furthermore, this method necessarily misses any CSF produced in the fourth ventricle and likewise the extra-choroidal CSF sources that do not drain into the lateral or third ventricles. As with the indirect method, the possibility cannot be ignored that traumatic injury inflicted by cannula insertion may perturb CSF production.

A modified approach for directly measuring CSF production was first developed in anesthetized rats [82, 83]. In this procedure, a 23-gauge needle connected to a PE-20 tubing is inserted into the fourth ventricle from the cisterna magna and used to infuse mineral oil (100 μL) to block the aqueduct of Sylvius. Meanwhile, the left lateral ventricle is cannulated using a pre-marked glass capillary tube*.* The CSF production is recorded through marking the position of the fluid lemniscus within the tubing at five- or ten-minute intervals. The CSF production rate is then calculated by using the formula:

$$V_{f} = \pi R^{2} \cdot{\text{L,}}$$

$V_{f}$: CSF production rate, $R$: the radius of the PE tubing, ${\text{L}}$: the length of CSF outflow in a given time

**Fig. 8:**
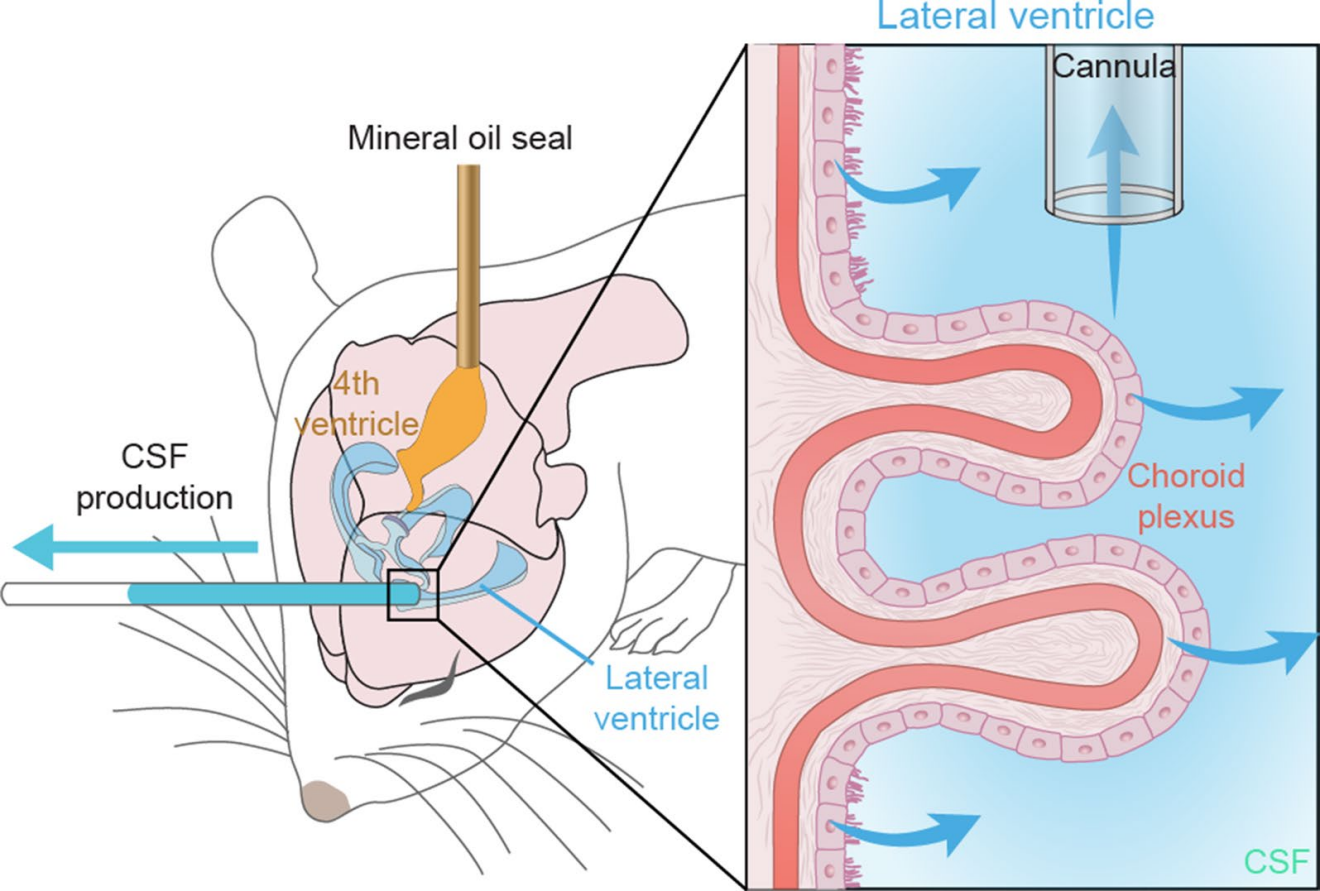
Direct CSF Production Measurement in rodent. A lateral ventricle is cannulated with a glass capillary tube, while a needle is connected to tube inserted into the fourth ventricle and used to infuse 1 μL mineral oil to block the aqueduct of Sylvius. The volume of CSF production is measured from the tube in the lateral ventricle, using the measured volume-time relationship. Modified from Liu et al. [66]

Using this approach, Karimy et al. showed that CSF production is increased in a post-hemorrhagic model of hydrocephalus [82, 83]. Liu et al. [66] adapted this same methodology with blockage of the aqueduct of Sylvius to measure CSF production in mice. As depicted in Fig. 8, a 30-gauge needle connected to a PE-10 tubing is inserted into the cisterna magna and advanced two mm through the foramina into the fourth ventricle. A tiny volume (1 μL) of mineral oil is then infused to block firmly flow out of the 4th ventricle. Under these circumstances, CSF outflow will be forced to exit through the cannula placed in the lateral ventricle. The CSF production is measured and calculated by marking the position of the CSF lemniscus in the PE-10 tube every ten minutes, similarly to the approach used in rats [82]. Using this methodology, Liu et al. [66] showed that CSF production in murine brain was increased under isoflurane anesthesia as compared with ketamine/xylazine. Other pharmacological studies showed that pan-inhibition of adrenergic receptors modestly increased CSF production in awake mice. Conversely, aged mice exhibited a declining rate of CSF production, which was even further reduced in aged Alzheimer’s disease model mice overexpressing amyloid-β. Unexpectedly, and for as yet unexplained reasons, CSF production in young female mice was higher than in age-matched males. Yet, the direct method used by Karimy and Liu suffers from the shortcoming that in only collected the CSF produced by the CP of the lateral and third ventricle and extrachoroidal sources draining into the same ventricles. CSF produced in the fourth ventricle or from extra-choroidal sources not draining into the lateral and third ventricles is missed, leading to underestimation of the true production rate. The direct method has the advantage that it is relatively non-invasive and can be used in awake behaving mice.

#### Human

**Fig. 9:**
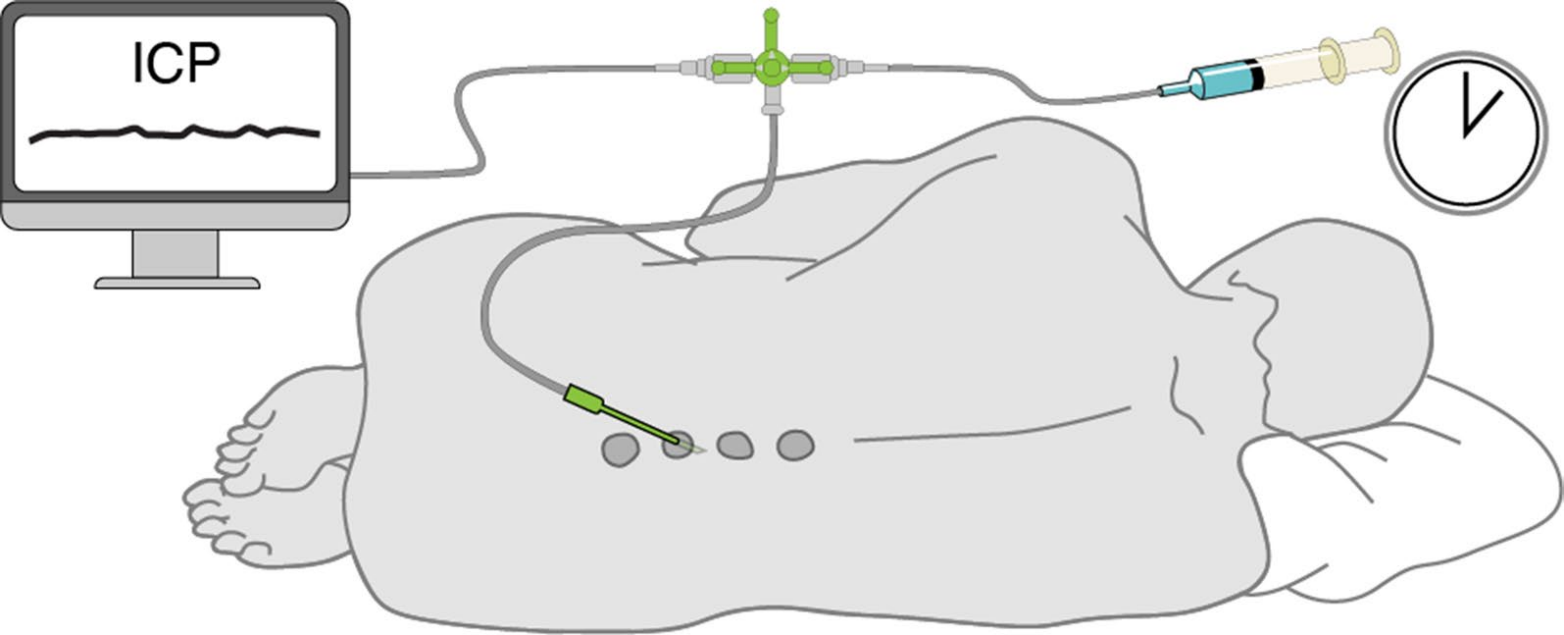
Illustration of the set-up for the CSF withdrawal in human. The patient is placed in the lateral recumbent position and a lumbar needle is inserted into the subarachnoid space. A certain volume of CSF is withdrawn through the needle, and ICP is monitored through a three-way stopcock. ICP will decrease during the acute CSF withdrawal, and the time required for the return of CSF pressure to its initial pressure is recorded to calculate the CSF production rate

Though a direct measurement of CSF production rate in humans had been obtained by the drainage method, the first precise and quantitative measurement was achieved by Masserman [84] in patients who required lumbar puncture for diagnostic or therapeutic purposes. The patient was placed in a comfortable lateral position, and a conventional lumbar puncture was performed with the needle inserted between the spines of the fourth and fifth lumbar vertebrae. A manometric apparatus was connected the lumbar puncture via a three-way valve (Fig. 9). A certain amount of CSF was withdrawn, and the time required for the return of the CSF pressure to its initial pressure was recorded. The CSF production rate was calculated by using the formula:

$$V_{f} = dV/T_{r} ,$$

$V_{f}$: CSF production rate, $dV$: the amount of CSF drained, $T_{r}$: the time required for the restoration of the pressure

Among the patients in whom this study was conducted, the calculated rate of CSF production following removal of 20–35 mL of CSF was 0.319 mL/min. However, after aninterval for recovery, the measurement was repeated following the identical procedure, giving a follow-up production rate 0.376 mL/min, corresponding to an 18% increase in the production rate [84]. Thus, CSF production may increase acutely in response to demand. This method can be used safely in humans, thus averting the need for invasive operative procedures, as in the indirect (perfusion) method. The resultant CSF production rate (~ 0.4 mL/min) thus exceeded the roentgenographic method (0.07 mL/min) [42], and matching that from the indirect method (0.35–0.37 mL/min) [75, 77]. This methodology has been used to evaluate the production rate of CSF in, for example, Down’s Syndrome individuals [85], and healthy aged subjects [86]. Silverberg et al. [25] later modified this approach to access the ventricles rather than the lumbar subarachnoid space. He removed only 3 mL of CSF rather than the minimum 10 mL volume required for tapping the lumbar subarachnoid space [25].

However, we note several limitations associated with the above procedure: (1) there is a risk for neurovascular congestion and edema after lumbar puncture and rapid drainage of CSF [87]. The rapid removal of more than 10 mL CSF can provoke a vascular dilatation in the central nervous system, and the rapid drainage of more than 35 mL may result in shrinking of the ventricular and subarachnoid spaces, which can begin immediately after the drainage and persist for eight hours or longer [84]. (2) Absorption and efflux of CSF are likely to continue during the measurement, leading to an underestimation of CSF production rate. (3) The compliance of the subarachnoid space in response to changes in vascular volume might result in erroneous values of CSF production. In particular, draining CSF from the subarachnoid space will lower the pressure of that compartment, resulting in lesser absorption of CSF into the superior sagittal sinus [88].

### Phase-contrast magnetic resonance imaging (PC-MRI) method

**Fig. 10:**
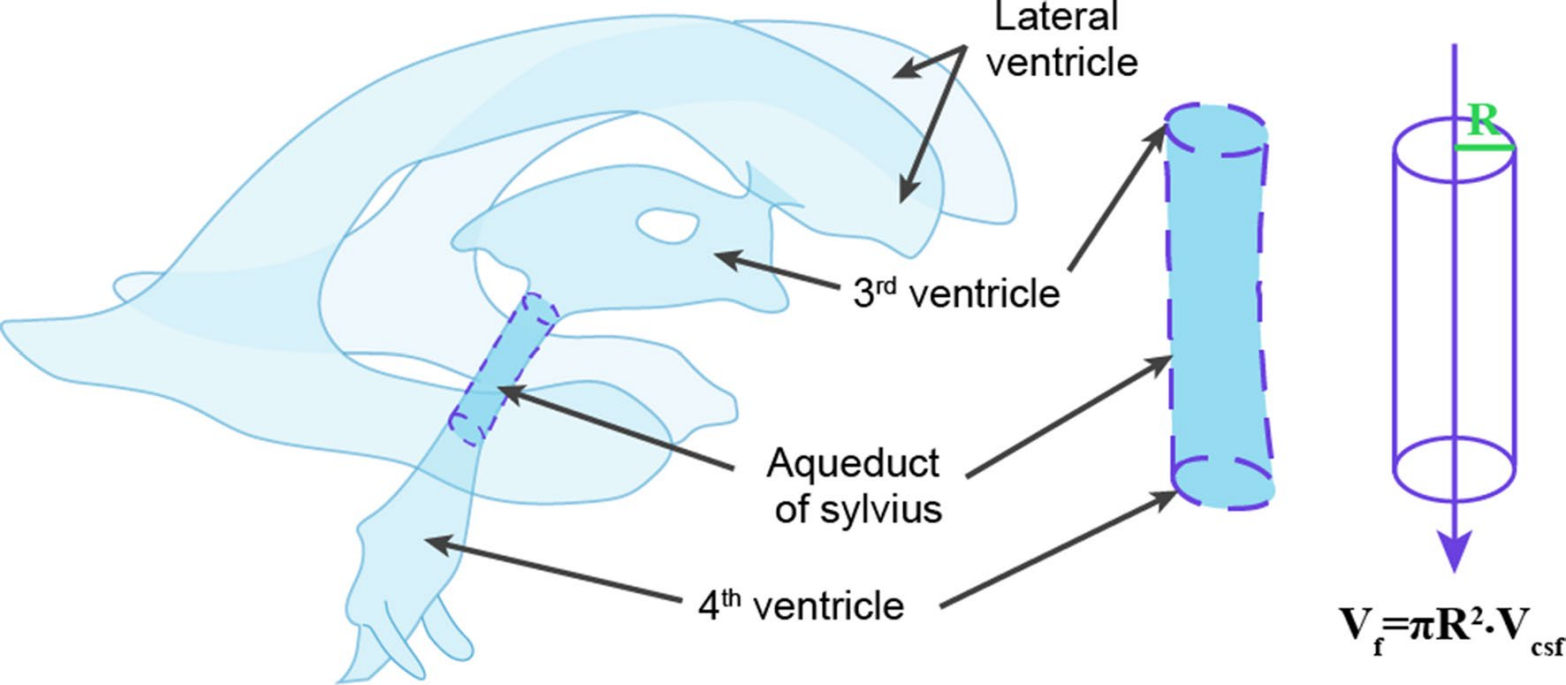
Scheme of the human brain CSF system. The velocity of the CSF per unit time within the aqueduct is measured by the phase-contrast magnetic resonance imaging (PC–MRI) method. Inset is the aqueduct of Sylvius, which is considering to be a cylinder through which the CSF passes. The CSF production rate is calculated using an equation based on CSF velocity using the MRI method

Magnetic resonance imaging (MRI), more specifically phase-contrast MRI (PC-MRI), is a noninvasive method that can be used to study the dynamics and flow pattern of CSF in the central nervous system of awake individuals. The PC-MRI sequence is a combination of imaging two tools, namely phase contrast and cardiac cine MRI, as first described by Nayler et al. [89]. This method generates a signal contrast between flowing and stationary hydrogen nuclei by sensitizing the transverse magnetization phase to the velocity of motion [90–95]. This method was first used to study the direction and velocity of blood flow [96–99]. Feinberg et al. [100] then developed a new MR imaging sequence for quantitative measurement of CSF velocity and brain motion in awake human subjects. Through an assessment of CSF flow along the aqueduct connecting the third and fourth cerebral ventricles, Feinberg et al. made an important proof of principle for PC-MRI technology. Linninger et al. [101] then applied this technology to measure accurately the CSF flow velocities in selected regions parts of the human ventricular system, and Wymer et al. [45] subsequently managed to generalize its use in other organs. An underlying condition for its application in the brain is to model the aqueduct of Sylvius as a cylindrical tube through which CSF flows, comparable to the blood flow through arteries. The average diameter of the aqueduct in human adult brain is only 2.0 mm [36, 102]. If one measures the diameter of the lumen in structural images and the flow rate of CSF per unit time, then the CSF production rate is then calculated as follows (Fig. 10):

$$V_{f} = \pi R^{2}\cdot{ }V_{csf} ,$$

$V_{f}$: CSF formation, $R$: the radius of the aqueduct, $V_{csf}$: the actual flow rate of CSF [36]

Given its noninvasive nature and its compatibility with physiologically normal conditions, MRI is the only approach to study regional CSF dynamics in the human brain. However, the quantitative results for MRI can deviate importantly from other estimates. Enzman and Pelc measured a net flow rate in the aqueduct consistent with other results, but also noted temporal dynamics such that net CSF flow during a single cardiac cycle was too small to detect [103]. Other groups found aqueduct flow rates in healthy adults ranging from 0.3 to 1.2 mL/min [104–106], with a mean of around 0.7 mL/min [107, 108]. One publication reported caudo-cranial net CSF flow in the cranio-cervical junction and cerebral aqueduct in idiopathic normal pressure hydrocephalus (iNPH) patients and healthy subjects using a detailed pixelwise PC-MRI analysis [109]. Results showed a CSF volumetric net flow rate of 3.125 mL/min in the cranio-cervical junction, thus greatly exceeding the 0.205 mL/min flow in the cerebral aqueduct was in healthy individuals. Although the PC-MRI method is now widely accepted for studying CSF dynamics in humans, there are some concerns about its accuracy that arise from certain limitations of this method. For instance, the PC-MRI approach does not include the CSF volume produced in the CP within the fourth ventricle, nor does it accommodate the CSF volume generated from extra-choroidal sources except those draining into the lateral and third ventricles. However, CSF production also arose from the spinal canal or caudal to the craniocervical junction in healthy controls and iNPH patients [109]. Another research group found that CSF in the spinal canal moves upward toward the head in response to forced inspiration [110]. They detected an increase of the venous outflow from the head in epidural veins located at the level of the third cervical vertebra that accompanied the reduction of intrathoracic pressure during deep inspiration. At the same time, they saw an increased CSF flux directed upward in the upper cervical and upper middle thoracic spine, and in the aqueduct. They speculated that the upward CSF flow and the enhanced venous outflow may be mutually compensatory phenomena, following the Monro–Kellie doctrine that the intracranial volume is constant.

A second caveat about the PC-MRI methods is that, applying the above formula, the aqueduct is regarded as being a cylinder, although structural investigation reveals a typically triangular profile of the aqueductal adytum [111], with different segments having different diameters. If the diameter of the aqueduct actually exceeds 1.5 mm [102], the use of MRI to measure the flow rate is considered feasible, but the method’s accuracy is compromised for channels of smaller diameter, and the method is unfit for experimental animals of small brain weight. A third issue concerns the effects of respiration and cerebral vascular pulsation on the relative position change of the aqueduct, which may cause a shift in the region of interest (ROI) and partial-volume effects, thus introducing variability and calculation errors if such shifts and partial-volume effects are not taken into account [112]. Fourth, the mean velocity measured by PC-MRI is used to calculate the CSF flow rate, with averaging of the PC-MRI acquisitions over a large number of cardiac cycles. Consequently, the final velocity waveform represents an average measurement for many cycles [113]. According to the theoretical assumption the aqueduct is a cylindrical tube, the acquisition is performed perpendicular to its supposed cross-sectional area. This approach assures that mainly the signals from the inflowing protons are detected, while minimizing the partial volume effects from the static protons [114]. As such, the instantaneous flows both forward and backwards in aqueduct can be measured with considerable accuracy. Although net flows are difficult to estimate with high reliability, the method is fit to depict instantaneous changes in flow rate [112].

A fifth caveat is that the size of the aqueduct has been reported to vary across the cardiac cycle, which introduces bias into calculation of the CSF production rate [115]. Sixth, results from multiple MRI techniques have shown that CSF flow is not unidirectional, but includes a substantial retrograde flow [116–118]. Furthermore, CSF flow in the ventricular system and subarachnoid space may large consist of irregular motion, which is not represented in the model [119]. Finally, net CSF flow through the cerebral aqueduct increases during expiration and reverses in direction during inspiration, such that net CSF flow measurements with PC-MRI are not sufficient to quantify total CSF production [120]. Despite all of these potential caveats, PC-MRI is a generally accepted approach, and most data in the literature on human CSF production is based on this methodology. However, as described below, rapid developments in MRI technology are poised to supplement the CSF production data obtained with PC-MRI.

### Time–spatial labeling inversion pulse (Time-SLIP)

**Fig. 11:**
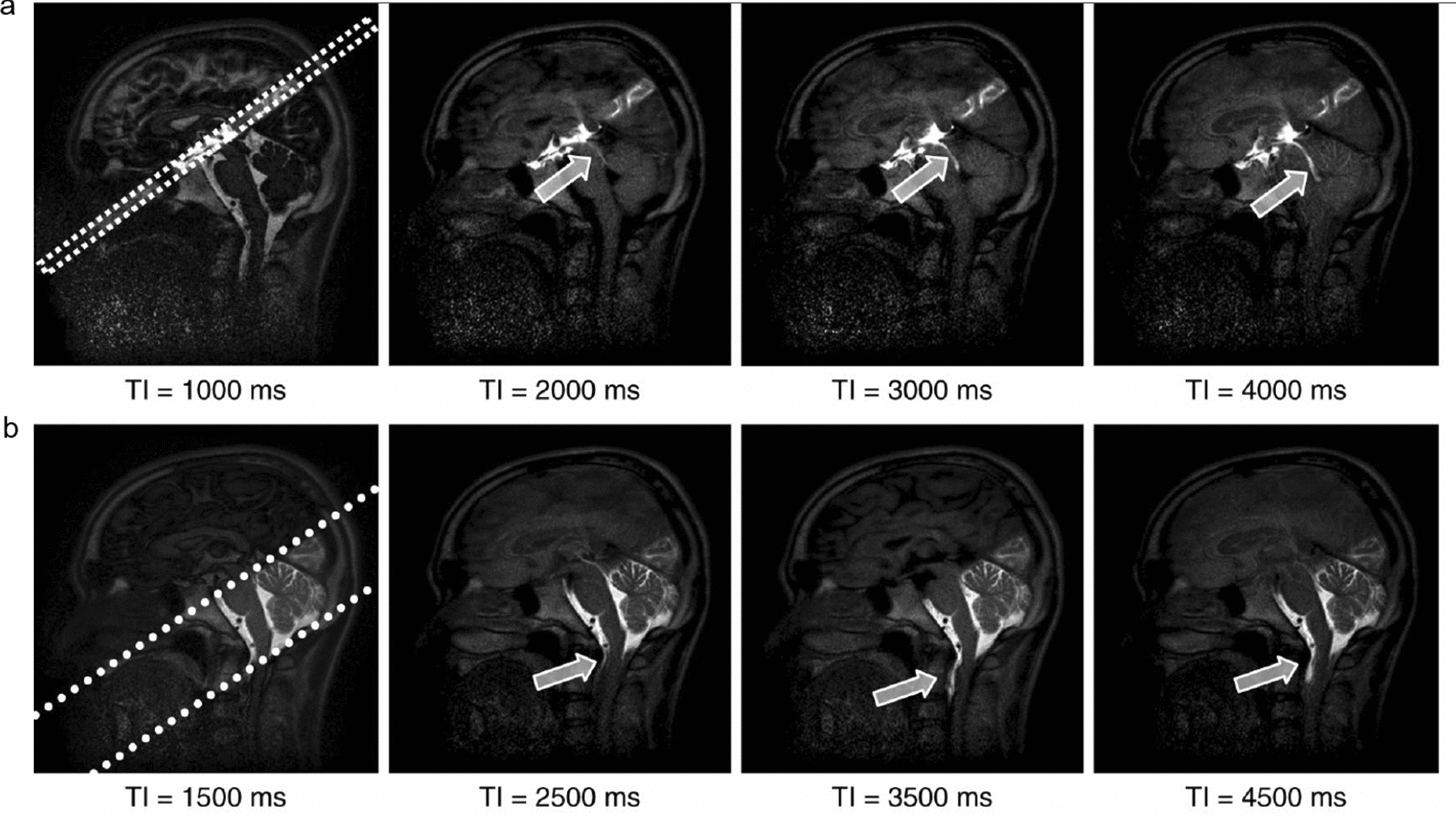
Using the time-spatial labeling inversion pulse (SLIP) technique to detect CSF movement. **a** Images of pulsed CSF at the indicated inversion time (TI). The labeling pulse is 1 cm thick (dotted lines) and is applied at a right angle in a region of interest that covers the third ventricle. The nonlabelled CSF signal at TI between 2000 and 4000 ms is null, but the pulse-labeled CSF shows high signal intensity during the same period. **b** The movement of CSF from the prepontine subarachnoid space into the spinal subarachnoid space through the cisterna magna, as indicated by arrows, can be seen in a much wider labeled section (dotted lines) that covers the posterior fossa. From Yamada et al. [121]

Yamada et al. have used the Time-SLIP technique in which CSF is spin-tagged and thereby act as an endogenous tracer to noninvasively visualize CSF bulk and turbulent flow without invasive administration of a contrast agent (Fig. 11) [121]. This approach acquires a series of single-shot images with incremental inversion recovery times, allowing the linear and turbulent movement components of CSF to be seen for up to five seconds [113]. Using a 2D fast advanced spin-echo sequence as the fundamental acquisition scheme, Time-SLIP can depict the movement of CSF in small incremental steps, independent of the cardiac cycle, and show how CSF flow patterns are altered by the extent of respiratory effort [113].

The Time-SLIP technique can be used to assess CSF movements in normal physiological and in pathological conditions, with labeling of a selected volume of CSF in any part of the brain and spinal cord. For example, on one application of Time-SLIP served to test whether the two CSF cistern are connected [121]. The investigators consistently found turbulent CSF flow from the aqueduct to the third ventricle, but bulk CSF flow from the lateral ventricles through the fourth ventricle to the spinal subarachnoid space in a group of healthy volunteers. The study thus confirmed the expected bidirectional exchange of CSF between the third and lateral ventricles [121]. The same research group also used this method in patients with hydrocephalus, arachnoid cyst and symptomatic cavum septi pellucidi, and compared preoperative and postoperative reflux flows [121, 122]. However, there are problematic issues that must be considered when using the Time-SLIP method: (1) Although this technique can display details of CSF including reflux and turbulence, it cannot yet support quantitation of CSF production; (2) Since there is a 2D labeling pulse in the selected section, the best view of CSF movement is in the labeling section perpendicular to the direction of CSF flow, and is only technically possible with an inversion time of 1500–4500 ms [113].

### Arterial spin labeling magnetic resonance imaging (ASL-MRI)

**Fig. 12:**
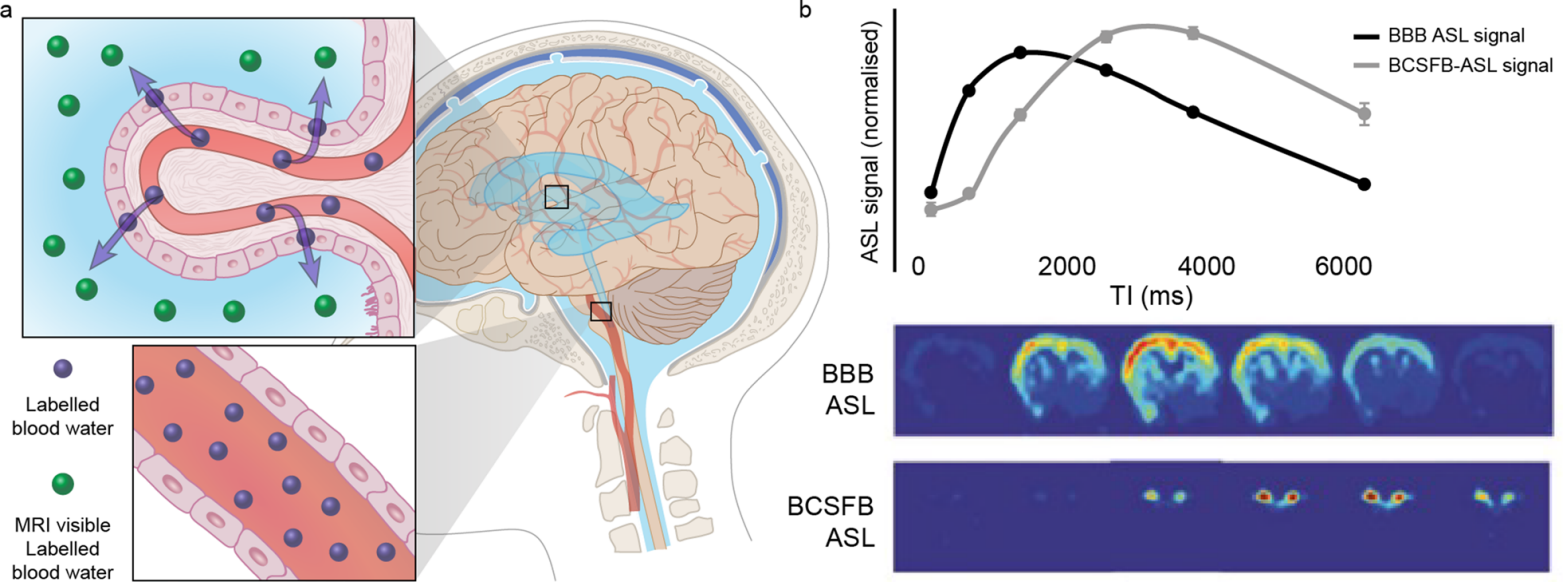
Non-invasive MRI of the blood–cerebrospinal fluid barrier (BCSFB) function. **a** Diagram for the principle underlying arterial spin labeling. The arterial blood water of the brain feeding arteries is spin-labelled, and the signal is measured from labelled blood water that has been transported to the CSF. **b** Above—traditional arterial spin labeling (ASL) signal probes blood–brain barrier (BBB) function and the novel BCSFB-ASL signal, below—ASL images from a single mouse. Modified from Evans et al. [130]

Arterial spin labeling (ASL) is a non-invasive MRI technique that entails magnetically uses arterial blood water as an endogenous tracer to map and quantify cerebral blood flow [123–129]. The method was originally developed Williams et al. to measure the blood flow in rat brain, and proved sensitive to detect regional abnormalities in rat cerebral perfusion [125]. The ASL approach was later modified to study blood flow in the human brain [127]. The underlying principle is to spin-label blood water in the brain’s feeding arteries and then measure the concentration of spin-labeled water in the ventricles, as first introduced by Evans et al. [130]. Traditional ASL sequences include a short wait (post-labeling delay, PLD) between labeling and imaging to allow the labelled water to reach the tissue compartment. After this delay, Petitclerc et al. found ASL signal both in the ventricular choroid plexus and the subarachnoid space, thus demonstrating that a considerable amount (~ 20%) of the labelled blood water is delivered to the CSF, and that the blood-CSF water exchange time was relatively fast, in the range of 50–70 s [131]. Several investigators have improved the techniques for detecting the movement of water by prolonging the labelling duration times and PLD time. Zhao et al. [132] used a very slow T_1_ decay of CSF (∼ 4300 ms) to build up the labeled signal for a longer time. By extending the TE (e.g., 220 ms at 9.4 T), CSF-derived signal can be isolated from other perfusion or faster/turbulent flow compartments within the brain tissue (Fig. 12) [130, 133]. Wells et al. [134] developed a multiple echo time (multi-TE) ASL-MRI technique, which is able to estimate rates of labelled vascular water delivery across the blood–brain interface in the mouse brain parenchyma [135], as a surrogate index of BBI permeability to water. Based on the underlying theory, a multi-time-point, multi-echo ASL protocol [131] was used to measure the amount and location of water transport across the blood-CSF barrier (BCSFB) in the human brain, and to model and characterize the dynamics of blood-CSF water exchange. In a pharmacological challenge study using ASL-MRI, systemic administration of anti-diuretic hormone (vasopressin) significantly reduced both CP blood perfusion and BCSFB water flow, which validates the claim that BCSFB water flow is a metric of choroidal CSF secretory function [136]. Vasopressin has previously been shown to reduce CP blood perfusion in rabbit experiments using the invasive microsphere technique [137]. Results from the (multi-TE) ASL-MRI technique [130, 131] confirmed that: (1) the novel BCSFB functional signal derives from endogenous arterial blood water that has been delivered to ventricular CSF; (2) there are exchange sites of CSF-ASL signal that lie outside the CP in the human brain, whereas the CSF-ASL signal is concentrated in the CP area [45]; (3) the CSF signal can be isolated from other compartments based on its distinctly prolonged T2 time; (4) water in the vascular compartment may also pass-through the brain blood barrier (BBB) to form CSF, thus constituting an extra-choroidal source of CSF [14, 138, 139]. Taking the average rate of BCSFB-mediated labelled water delivery to the lateral ventricles, and considering the total size of the functional voxels (11.25 mm^3^) returns a total BCSFB-labelled water delivery rate to the lateral ventricles of 2.7 µL/min in mouse brain [130]. Unsurprisingly, this is markedly greater than previous estimates of CSF secretion in murine brain (~ 0.3 µL/min), which doubtless reflects the higher rate of water exchange/flux as compared to net secretion across blood vessels [140].

Notwithstanding these results, there are some concerns about the accuracy and the generalizability of the ASL-MRI technique: (1) The rate of labelled water passing from the CP to the ventricles reflects the permeability of water across the BCSFB, not CSF production per se; (2) the observations are quantitative for net rates of water delivery from arterial blood to ventricular CSF, but not in terms of volume. As such, the BCSFB-ASL result represents a correlate of CSF secretion and indicates the expected direction of the water exchange between the two compartments [130]. As an indirect measure of CSF production, BCSFB-ASL can detect relative changes in CSF production. It should however be noted that several recent publications have suggested that the Na^+^-K^+^-2Cl^−^ cotransporter 1 (NKCC1) expressed by choroid plexus endothelial cells acts as a buffer of K^+^ and thereby of active water uptake, during both neurodevelopment and in the adult brain [141, 142]. BCSFB-ASL can only detect unidirectional fluid secretion and will not account for possible fluid uptake by the CP; (3) it is assumed that the labeled water enters only the CSF and no other compartment; (4) the blood-CSF exchange is assumed to be unidirectional rather than bidirectional, thus neglecting the exchange of CSF back to the blood; (5) Evans et al. demonstrated that the BCSFB-ASL signal co-localizes with the CP within the lateral ventricles, and that the CSF-ASL signal was concentrated in the CP area [130]. The authors observed no CSF-ASL signal outside of the CP in mice. However, Petitclerc et al. found the signal to be widely distributed, and also detectable in human cerebral cortex [131]. This discrepancy could be due to the low subarachnoid-CSF volume in mice in conjunction with limited image resolution of the method. Alternatively, differences in the polarization of aquaporin water channels between humans and mice, as previously shown for AQP-4 in astrocytes [143], might affect the ability of transfer the fluid in the brain. Recently, a CSF-ASL signal was detected in rat cerebral cortex by using interleaved short and long echo time continuous ASL acquisition [136]. Further study is needed to account for the lack CSF-ASL signal outside the CP in mice.

## Discussion

The study of CSF spans several millennia of recorded history (Fig. 1). The recent rapid development in our understanding of brain fluid flow has sparked a renewed interest in establishing how CSF is secreted, its ionic and molecular composition, and its rate of production and turnover. The CSF production rate in humans decreases with age [66], although the total CSF volume increases due to brain atrophy [144]. Thus, the CSF turnover rate, calculated by dividing the CSF production rate by the total CSF volume, declines substantially across the human lifespan [145]; daily turnover is 6 for newborns, 4.5 for young adults, and only 3 times a day for the elderly [86]. One might expect that the age-related decline in CSF turnover rates can be extended to reduced clearance of metabolic waste products and neurotoxic solutes such as amyloid-β. In fact, CSF production rate is suppressed in transgenic Alzheimer’s disease model mice [66] and in patients with moderate-to-severe Alzheimer’s disease compared to healthy aged controls [25]. Taken together, an emerging literature suggests that declining CSF turnover may contribute to neurodegenerative diseases.

As discussed in this review, many different methods have been developed to measure CSF production in different species (Table 1), dating back nearly a century. In experimental studies, the indirect method (the tracer dilution method) developed by Pappenheimer [40] has historically been the most frequently used. The indirect technique was first used in goats with chronically implanted intraventricular and intracisternal cannulas. Later, the indirect CSF production technique was adapted for use in several mammalian species, including marine animals [146]. According to this method, the CSF production rate was ~150 μL/min in goats [40], as later confirmed by other groups [37, 59]. The use of the indirect method is associated with considered variability, which may arise due to a number of factors. For example, the tracer may pass through the ventricular wall and enter the surrounding brain tissue [66], thus leading to overestimation of the CSF production. Furthermore, the rate of infusion of aCSF can perturb CSF production. Indeed, faster infusion rates reduce the difference between the tracer concentration of infusion and outflow samples, thereby reducing the calculated CSF production rate [70].

One unexplained observation is that the indirect method consistently indicates a 30% higher CSF production compared to the direct method in dogs [147, 148], and 24% higher in cats [149]. For rats and mice, the value obtained by the indirect measurement method is threefold higher than according to the direct measurement method [66, 150, 151]. These discrepancies may be related to the direct measurement method missing any CSF produced in the fourth ventricle. The CP in the fourth ventricle in rabbits accounts for one third of the total weight, likely contributing to the substantial underestimation by the direct method [35]. Furthermore, CSF hyperproduction might be triggered by infusion of fluid into the lateral ventricles with the indirect method.

Alternatively, the contribution of extra-choroidal CSF production might be larger than is generally acknowledged, as suggested by several lines of evidence [38, 152, 153]. Indeed, a dural source of CSF was postulated by Weed as early as 1914 [154]. Weed suggested that CSF flows against gravity from the base to the top of the brain. He also suggested that osmosis and other physicochemical factors may result in tissue fluids carrying metabolic waste products away from sources of high neural activity towards the adventitial spaces of the walls of the blood vessels and capillaries, thus predicting the glymphatic concept. Hassin went further to propose in the 1920 s and 30 s that CSF represents the tissue fluid of the brain, and that CSF in the subarachnoid space is a derivative of tissue fluids produced by the cerebrospinal parenchyma [155, 156]. In one early experiment, complete surgical removal of CP did not ablate CSF production [157]. Wallace & Brodie demonstrated that anions entered CSF as readily from the parenchymal capillaries as from the ventricular choroid [158]. Boldrey et al. showed production of CSF in the spinal subarachnoid space as well as in the ventricles in humans [159]. Furthermore, Sweet and Locksley proved that water and electrolytes can move between CSF and blood, both in the ventricular system and the subarachnoid space [160]. The most common estimate is that ~ 80% of the CSF is secreted by the CP, where the remaining ~ 20% derives from the extra-choroidal tissue and the interstitial fluid, or by influx of vascular fluid across the BBB [152, 153, 161]. However, technological innovations may lead to a revision of this notion. We hold that none of the existing techniques for measurement of CSF production are ideal or beyond reproach. The rate of extrachoroidal CSF production derived from capillary fluid influx will be determined by the balance between hydrostatic and colloid osmotic pressure gradients. Any method that artificially alters hydrostatic or osmotic pressure will therefore be unfit to quantify absolutely the total rate of CSF production. Also, any method that does not include extra-choroidal sources of CSF will underestimate the total rate of CSF production.

Due to its invasiveness, use of the indirect perfusion method in humans has been limited to intraoperative studies in patients with CNS neoplasms such as gliomas or meningeal leukemia [75, 76]. MRI platforms may present the ideal approach to study CSF dynamics in the human CNS, as such methods are noninvasive and maintain normal physiological conditions. The principle of PC–MRI is that, under the effect of an external gradient magnetic field, static protons do not produce phase changes; only mobile protons produce phase changes, and consequently there arises a phase difference between mobile and static protons [45], which produces useful signals. These MRI-based methods were originally applied to study the velocity of blood flow, and are now by far the most widely used technique for human CSF research [45, 113, 162–165]. However, the quantitative results vary considerably in the range from 0.3 to 1.2 mL/min (Table 1) [104–107]. PC-MRI does not detect CSF produced in the fourth ventricle and extra-choroidal sources that do not drain into the lateral and third ventricles. Since invasive methods are likely to negatively impact CSF production, non-invasive approaches such as PC-MRI are to be preferred. However, it is important to note that PC-MRI is unfit for small-brained animals, and can only provide data on relative changes in CSF production. Several studies using different MRI approaches have been employed to understand what drives CSF flow. These studies have examined effects of cardiac [166, 167] and respiratory motion [110, 133, 168–170], and concluded that CSF motion in the spine and brain is modulated both by cardiac and respiratory motions. The amount of CSF displacement was larger in the respiratory component than in the cardiac component, both in the cranial and caudal directions. The movement of CSF due to the cardiac component was rapid and of small magnitude, while CSF movement due to the respiratory component was slow and large [118]. A comparable contribution of respiration and cardiac pulsations on CSF velocity was also found during deep breathing but not during natural breathing [171]. This impact of respiration on CSF flow is supported by computational studies [172].

Fultz et al. demonstrate that the sleeping brain exhibits waves of CSF flow on a macroscopic scale, and that these CSF dynamics are interlinked with neural and hemodynamic rhythms [173]. Thus, sleep can affect CSF flow. MRI-based methods applied in awake volunteers showed a nightly peak of 0.7 mL/h at 2:00 a.m., versus an evening nadir of only 0.2 mL/min at 6:00 p.m. [174], indicating that CSF production might also be under circadian control. The latter result was extended in a study by Hablitz et al. showing that glymphatic activity is regulated by the circadian clock [175]. Kiviniemi’s group used magnetic resonance encephalography imaging to show a clear difference in fluid flow in human brain in NREM sleep versus wakefulness and that slow vasomotion is a key driver of parenchymal fluid flow [176].

Sartoretti et al. used the PC-MRI technique to study the influence of age and sex on CSF flow dynamics parameters at the level of the intercollicular sulcus within the cerebral aqueduct [177]. Their study showed that most of flow parameters, including stroke volume, forward flow volume, backward flow volume, absolute stroke volume, mean flux, peak velocity, and peak pressure gradient, were significantly impacted by sex and age. The authors concluded that CSF volume regulation and flow may be impacted by hormones and neural systems. Indeed, using the direct method for quantifying the CSF production rate, we unexpectedly found that CSF production is higher in female than in male mice [66].

Classical theory of the “third circulation” holds that the CSF is mainly produced by the CP of the brain and subsequently leaves the ventricles via the foramen of Magendie and Luschka [34]. However, application of PC-MRI technology in vivo has led to a revision of this classic hypothesis. For example, CSF movement within the aqueduct is oscillatory and bidirectional and follows cardiac cycle-related cerebral blood volume variations [178–181]. PC-MRI methods have shown net retrograde flow in patients with iNPH which is not compatible with the third circulation concept [109, 182, 183]. Another study showed antegrade flow in the Sylvian aqueduct in healthy volunteers (though with a high degree of variability), which it was reversed in iNPH subjects [109]. Thus, the concept of the third circulation is currently debated in both physiological and pathological conditions. PC-MRI studies have provided evidence for CSF flow variability in patients with CSF disorders, including iNPH [184], communicating hydrocephalus [185], spontaneous intracranial hypotension [186], arachnoid cyst, pineal cyst [119], and idiopathic intracranial hypertension [187]. Thus, considerable quantitative in vivo evidence points towards substantial variations in the direction and magnitude of CSF flow in healthy controls and in subjects with CSF diseases [185, 188].

More recently, several imaging studies have used gadobutrol, a hydrophilic MRI contrast agent, as a CSF tracer to visualize the movement of CSF [189, 190]. Standardized T1-weighted MRI scans performed before and after intrathecal gadobutrol administration are used to follow CSF flow, which should in principle provide an indirect measure of CSF motion in real-time [185, 190]. Although gadobutrol has a small molecular weight (450 Da), it is still order of magnitudes larger than water (H_2_O, MW 18 Da), such that its transport may not capture all aspects of CSF flow [185]. In fact, a positron emission tomography (PET) study of CSF flow based on cisterna magna injection of H_2_^17^O provided evidence for a much more rapid CNS distribution of the radioactive water than for the heavier MR contrast agent Gd-DTPA (Magnevist; MW 938 Da). However, we noted that H_2_^17^O is not confined to the CSF compartment, but can freely pass the BBB and enter the blood pool [191]. In the H_2_^17^O PET study, muscle tissue served as a control to correct for BBB efflux. However, blood flow in resting muscle is much lower than in brain tissue in anesthetized non-moving animals. Ringstad and colleagues used intrathecal administration of gadobutrol as a tracer in patient with iNPH to study CSF and glymphatic function [189, 192, 193]. A series of experiments they proved the effectiveness of contrast-enhanced imaging to study CSF and glymphatic flow and added key information to the field.

Numerous studies have used pharmacological manipulations to study the mechanisms of CSF production in experimental studies. These studies have shown that treatment with ouabain, a Na^+^/K^+^-ATPase inhibitor, suppresses CSF production by 40–100% [48, 194, 195], whereas furosemide, an unspecific NKCC1 inhibitor, inhibited CSF secretion to a variable extent [196]. The Cl^−^ transport inhibitor DIDS was also reported to reduce CSF production rate [197]. In inflammatory conditions, a modelled in rats with intraventricular hemorrhage, hypersecretion of CSF was reported to be mediated by TLR4-dependent activation of the Ste20-type stress kinase SPAK, which binds, phosphorylates, and stimulates the NKCC1 at the choroid plexus epithelium apical membrane [83]. Overall, studies of the regulation of CSF production rate are technically challenging, because the pharmacological and genetic interventions might interfere with basic physiological parameters, such as blood and intracranial pressure, and thereby indirectly modulate CSF production [15]. ASL-MRI technology was initially used to study the cerebral blood flow [123–129]. The approach has now been expanded to include ASL surrogate measures of CSF secretion. Current ASL-MRI techniques detect unidirectional CSF outflow from the CP, but have not so far given accurate quantitation of CSF production, although we expect this limitation to be overcome by new technical innovations [130, 131, 136, 198]. By prolonging and changing the labelling duration times and PLD time, the movement of labelled water from the blood into the CSF through the CP can now be detected. Unlike in conventional ASL methods, Evans et al. have developed a method specifically designed to measure CP function in the lateral ventricles by using an ultra-long TE to ensure that only signal from the CSF is measured in the control/labelled images, through measurement of the BCSFB function in each ventricle [130]. Thus, this ASL signal may represent a meaningful and practical measurement index of ‘global’ function of the CP. By measuring CP perfusion and/or rates of labelled blood water delivery to ventricular CSF via the BCSFB, ASL-MRI techniques may come to provide a useful non-invasive approach to map and quantify CSF secretion across aging and in various disease conditions.

## Conclusions

There is burgeoning interest in the physiological regulation of CSF production and in how aging and diseases may affect CSF production. We have described in detail the many methods that have been developed to measure the production of the CSF, giving particular attention to their various limitation. Within a given species, the methods give a wide range of quantitative results (Table 1). An ideal methodology for quantifying CSF production rates has not yet been developed, but among the presently available methods, the non-invasive ASL-MRI technique is particularly promising for future research in human subjects.

## Data Availability

Not applicable.

## Abbreviations

CSF: Cerebrospinal fluid
CNS: Central nervous system
CP: Choroid plexus
ICP: Intracranial pressure
aCSF: Artificial CSF
MRI: Magnetic resonance imaging
PC-MRI: Phase-contrast magnetic resonance imaging
iNPH: Idiopathic normal pressure hydrocephalus
ROI: Region of interest
Time-SLIP: Time–spatial labeling inversion pulse
ASL-MRI: Arterial spin labeling magnetic resonance imaging
ASL: Arterial spin labeling
BCSFB: Blood-CSF barrier
BBB: Brain blood barrier
PLD: Post-labeling delay
NKCC1: Na^+^-K^+^-2Cl^−^ cotransporter 1
